# A cross-sectional study of multidimensional psychosocial stress and depression risk

**DOI:** 10.3389/fnbeh.2026.1786960

**Published:** 2026-03-12

**Authors:** Lu Han, Xingyu Chen, Xinyu Li, Peiyun Zhang, Jinlan Jiang, Xinxuan Lyu, Yanbing Lu, Yuzhen Chen, Wei Jin, Lihong Li

**Affiliations:** 1 The Second Clinical Medical College of Zhejiang Chinese Medical University, Hangzhou, Zhejiang, China; 2 School of Traditional Chinese Medicine, Beijing University of Chinese Medicine, Beijing, China; 3 The Second Affiliated Hospital of Zhejiang Chinese Medical University, Hangzhou, Zhejiang, China

**Keywords:** BMI, cross-sectional study, depression, psychosocial stress, risk factors

## Abstract

**Objective:**

This study aimed to investigate the cross-sectional associations of multidimensional psychosocial stress and body mass index (BMI) with depression risk.

**Methods:**

In this cross-sectional study, 222 participants (123 with depression, 99 controls) completed questionnaires assessing depression (BDI-II), six domains of psychosocial stress (family, work, financial, academic, interpersonal, emotional), BMI, and lifestyle factors. Multivariable logistic regression was used to examine independent associations, with exploratory subgroup analyses by age and gender.

**Results:**

Multivariate analysis indicates that, family stress (OR = 3.47, 95% CI: 1.96–6.15), academic stress (OR = 1.96, 95% CI: 1.14–3.38), and interpersonal stress (OR = 2.34, 95% CI: 1.36–4.03) were independently associated with higher odds of depression. Higher BMI was associated with lower odds of depression (OR = 0.90, 95% CI: 0.85–0.96); however, this inverse association may be confounded by unmeasured factors such as antidepressant use and should be interpreted cautiously. An extreme association with alcohol abstinence (OR = 0.05) was based on a very small subgroup (*n* = 14) and requires cautious interpretation. Exploratory subgroup analyses suggested variations in these associations.

**Conclusion:**

Specific psychosocial stressors are associated with depression risk in this sample. The counterintuitive finding regarding BMI warrants investigation in studies controlling for medication use. The subgroup findings are preliminary and require replication in larger cohorts.

## 1 Introduction

Depression is a prevalent mental disorder, with its global incidence rising by approximately 26.3% since 2010 (GBD 2023 Disease and Injury and Risk Factor Collaborators, 2025), thereby emerging as a major public health concern (World Health Organization, 2017, 2024). The causes of depression are complex and cannot be fully explained by any single biological or environmental factor (Marx et al., 2023). At both psychosocial and physiological levels, psychosocial stress and body mass index (BMI) have gained attention as modifiable factors that may play significant roles in the onset and progression of the disorder (Thapar et al., 2012). Existing research has identified multiple psychosocial stressors significantly associated with depression risk, including family conflict (Li et al., 2025; Lagdon et al., 2014), occupational stress (Chireh et al., 2025; Lee et al., 2017; Yoo et al., 2016), low socioeconomic status (Davis et al., 2025), academic burden (Wang J. et al., 2025), emotional trauma (Kuzminskaite et al., 2021; Reisner et al., 2025), and insufficient interpersonal support (Clayborne et al., 2019). BMI has also gained attention as a physiological factor linked to depression. Some studies suggest that higher BMI may increase depression risk through mechanisms such as inflammation, hormonal changes, or social stigma (Tyrrell et al., 2019; Hendy et al., 2025; Wang X. et al., 2025). However, findings regarding this relationship are complex and inconsistent. Moreover, psychosocial stress and BMI are often closely interrelated. For instance, stress may lead to changes in eating behaviors, potentially confounding their respective effects on depression (Milaneschi et al., 2019).

Most existing studies have focused on examining the independent effects of single stressors or BMI alone. This preliminary exploratory study aims to assess how multidimensional psychosocial stress and BMI are associated with depression risk, with the goal of generating hypotheses for future large-scale investigations. Specifically, using a cross-sectional design, this study evaluates the independent associations of multidimensional psychosocial stressors across family, work, financial, academic, emotional, and interpersonal domains with depression risk. Furthermore, it examines whether BMI is independently associated with depression risk after controlling for these stressors and demographic variables. These findings may inform future research on integrated interventions for depression.

## 2 Materials and methods

### 2.1 Ethics statement

The study protocol was approved by the Ethics Committee of the Second Affiliated Hospital of Zhejiang Chinese Medical University (2021-KL-075-01). Written informed consent was obtained from all participants prior to their inclusion in the study. For participants under the age of 18, informed consent was provided by their legal guardians.

### 2.2 Patient cohorts

This cross-sectional study was conducted at the Second Affiliated Hospital of Zhejiang Chinese Medical University from February 22, 2022, to December 31, 2024. Participant screening followed a multi-step protocol: recruitment was carried out via promotional posters, social media advertisements, and referrals from clinical psychologists. Eligible participants were subsequently administered the Beck Depression Inventory-II (BDI-II). Further assessment and group allocation were performed by the researchers based on the predefined inclusion and exclusion criteria.

Inclusion criteria:

Depression group: (1) Prior diagnosis of depression; (2) BDI-II score ≥ 14; (3) Age 10–70 years; (4) Absence of communication barriers and normal literacy; (5) Informed consent provided by the participant or their legal guardian.

Control group: No history of depression and BDI-II score < 14. All other criteria matched those of the depression group.

Exclusion criteria: (1) History of other mental disorders; (2) Unreliable responses (identical answers across all items); (3) Questionnaires submitted from the same IP address or completed in less than 10 min.

**FIGURE 1:**
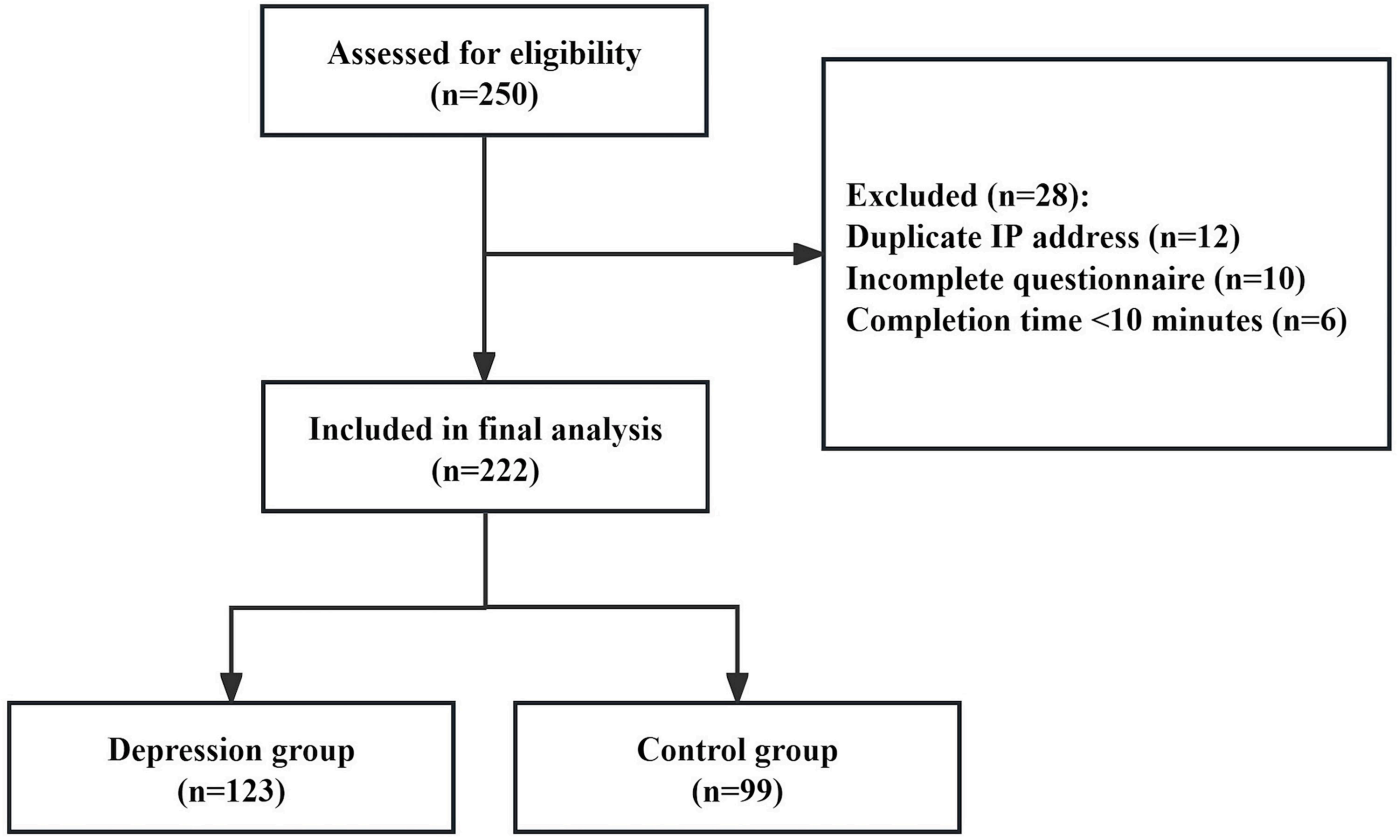
Participant flowchart. A total of 250 individuals were assessed for eligibility. Exclusion criteria were as follows: duplicate IP addresses (*n* = 12); incomplete questionnaire responses (*n* = 10); completion time less than 10 min (*n* = 6). The final analysis included 222 participants (depression group: *n* = 123; control group: *n* = 99).

A total of 250 individuals were initially assessed for eligibility. Among them, 28 were excluded for the following reasons: duplicate IP addresses (*n* = 12), incomplete questionnaire responses (*n* = 10), and completion time of less than 10 min (*n* = 6). Consequently, 222 participants were included in the final analysis, comprising 123 in the depression group and 99 in the control group. A participant flow diagram is provided in Figure 1.

### 2.3 Data collection and definitions

Depressive symptoms were assessed using the BDI-II, a 21-item self-report questionnaire with total scores ranging from 0 to 60 (Button et al., 2015). Depression was defined by a score of ≥ 14 on the BDI-II (Wang and Gorenstein, 2013), a tool with established reliability and validity in Chinese populations (Wang et al., 2020; Sun et al., 2017).

Demographic and clinical data were collected using a standardized questionnaire, including age, gender, BMI, occupation, smoking status [current, former (quit > 6 months ago), or never], alcohol use status [current, former (quit > 6 months ago), or never], and psychosocial stress. BMI was calculated from self-reported height and weight, using the formula BMI = weight (kg)/height^2^ (m^2^), and analyzed continuously to assess its association with depression.

Psychosocial stress was evaluated through six independent yes/no questions assessing whether participants had experienced significant stress in the following domains over the past year: family, work, finances, academics, interpersonal, and emotional stress. Each domain was assessed with a single direct question:

For family stress: “In the past year, have your family relationships or family matters caused you significant stress or distress?”

For work stress: “In the past year, have your work, employment, or job-related matters caused you significant stress or distress?”

For financial stress: “In the past year, have your personal financial situation or difficulties caused you significant stress or distress?”

For academic stress: “In the past year, have your academic workload, performance, or studies caused you significant stress or distress?”

For interpersonal stress: “In the past year, have your relationships with friends, colleagues, or others outside your family caused you significant stress or distress?”

For emotional stress: “In the past year, has your romantic or intimate relationship caused you significant stress or distress?”

Responses were coded into six binary variables (1 = presence of stress in that domain, 0 = absence) for subsequent analyses.

Based on previous literature, age, gender, smoking status, and alcohol use were included in the analyses as potential confounding variables.

### 2.4 Statistical analysis

All statistical analyses were conducted using R software (version 4.4.3). All collected questionnaires were examined for missing values, and only those with complete data across all analytical variables were included in the analysis.

For comparisons of baseline characteristics between the depression and control groups, continuous variables conforming to a normal distribution were expressed as mean ± standard deviation (Mean ± SD) and analyzed using independent samples *t*-tests. Categorical variables were summarized as frequency (percentage) and compared using chi-square (χ^2^) tests.

Given the limited sample size of this study, multivariable logistic regression was employed to assess the independent associations of psychosocial stress and BMI with depression risk. All six psychosocial stress domains (family, work, financial, academic, interpersonal, and emotional stress) were included as prespecified predictors based on the study’s aims. The final adjusted multivariable logistic regression model included the following variables: age, gender, BMI, occupation, smoking status, alcohol use status, and all six psychosocial stress domains. Results are reported as adjusted odds ratios (OR) with 95% confidence intervals (CI).

To further examine potential variations in the association between psychosocial stress and depression across subgroups, stratified analyses were performed by age (≤ 24 years vs. > 24 years) (Sawyer et al., 2018) and gender. A two-sided *P*-value < 0.05 was considered statistically significant. The subgroup findings are preliminary and should be viewed as exploratory, providing clues for future hypothesis generation rather than conclusive evidence.

The primary multivariable logistic regression model included 14 predictors (age, gender, BMI, occupation, smoking status, alcohol use status, and six psychosocial stress domains). With 123 events in the depression group, the events-per-variable (EPV) ratio was approximately 8.8. Although this falls slightly below the conventional threshold of 10 recommended for confirmatory studies, it is generally considered acceptable for exploratory, hypothesis-generating analyses (Peduzzi et al., 1996; Vittinghoff and McCulloch, 2007).

## 3 Results

### 3.1 Baseline characteristics

A total of 222 participants were included in this study, with 123 individuals (55.4%) in the depression group and 99 individuals (44.6%) in the control group. The baseline characteristics of both groups are compared in detail in Table 1.

**TABLE 1:** Comparison of baseline characteristics between the depression group and the control group.

Variables	Total (*n* = 222)	Non-depressed (*n* = 99)	Depressed (*n* = 123)	Statistic	*P*
Age, Mean ± SD	22.91 ± 10.73	23.39 ± 10.47	22.52 ± 10.95	*t* = 0.60	0.548
BMI, Mean ± SD	21.89 ± 4.96	23.13 ± 6.06	20.89 ± 3.58	*t* = 3.24	**0.001**
Gender, n (%)				χ^2^ = 3.50	0.061
Male	75 (33.78)	40 (40.40)	35 (28.46)		
Female	147 (66.22)	59 (59.60)	88 (71.54)		
Job, n (%)				χ^2^ = 1.18	0.277
Student	141 (63.51)	59 (59.60)	82 (66.67)		
Non-student	81 (36.49)	40 (40.40)	41 (33.33)		
Smoke, n (%)				-	0.154
No	208 (93.69)	94 (94.95)	114 (92.68)		
Yes	10 (4.50)	2 (2.02)	8 (6.50)		
Quit	4 (1.80)	3 (3.03)	1 (0.81)		
Alcohol, *n* (%)				χ^2^ = 14.09	**< 0.001**
No	198 (89.19)	82 (82.83)	116 (94.31)		
Yes	10 (4.50)	4 (4.04)	6 (4.88)		
Quit	14 (6.31)	13 (13.13)	1 (0.81)		
Family stress, *n* (%)				χ^2^ = 18.92	**< 0.001**
No	128 (57.66)	73 (73.74)	55 (44.72)		
Yes	94 (42.34)	26 (26.26)	68 (55.28)		
Work stress, *n* (%)				χ^2^ = 0.02	0.897
No	165 (74.32)	74 (74.75)	91 (73.98)		
Yes	57 (25.68)	25 (25.25)	32 (26.02)		
Academic stress, *n* (%)				χ^2^ = 5.94	**0.015**
No	90 (40.54)	49 (49.49)	41 (33.33)		
Yes	132 (59.46)	50 (50.51)	82 (66.67)		
Interpersonal stress, *n* (%)				χ^2^ = 9.48	**0.002**
No	118 (53.15)	64 (64.65)	54 (43.90)		
Yes	104 (46.85)	35 (35.35)	69 (56.10)		
Emotional stress,*n* (%)				χ^2^ = 0.36	0.551
No	180 (81.08)	82 (82.83)	98 (79.67)		
Yes	42 (18.92)	17 (17.17)	25 (20.33)		
Financial stress, *n* (%)				χ^2^ = 0.54	0.461
No	203 (91.44)	89 (89.90)	114 (92.68)		
Yes	19 (8.56)	10 (10.10)	9 (7.32)		

Categorical variables were compared using chi-square (χ^2^) test. *P*-values are provided for descriptive characterization of baseline differences only; all variables were retained in the multivariable model based on prespecified theoretical relevance. Bold values indicate statistical significance at *P* < 0.05.

Baseline comparisons showed no statistically significant differences between the two groups in age, gender, occupation, smoking status, work stress, emotional stress, or financial stress (all *P* > 0.05). In contrast, significant differences were observed in BMI (*P* = 0.001), alcohol consumption status (*P* < 0.001), family stress (*P* < 0.001), academic stress (*P* = 0.015), and interpersonal stress (*P* = 0.002).

### 3.2 Logistic regression analysis

Following the modeling strategy described in the Methods, the final adjusted multivariable logistic regression model was fitted. The results are presented in Table 2. The analysis showed that family stress, academic stress, interpersonal stress, BMI, and alcohol abstinence were significantly associated with depression (all *P* < 0.05). Among these factors, alcohol abstinence was associated with a markedly reduced risk of depression (adjusted OR = 0.05). The alcohol abstinence subgroup was small (*n* = 14).

**TABLE 2:** Multivariate logistic regression analysis results for factors influencing depression.

Variables	β	S.E	*Z*	OR (95% CI)	*P*
Age	−0.01	0.01	−0.60	0.99 (0.97–1.02)	0.546
BMI	−0.10	0.03	−3.14	0.90 (0.85–0.96)	**0.002**
**Gender**					
Male	−	−	−	1.00 (Reference)	−
Female	0.53	0.29	1.86	1.70 (0.97–2.99)	0.062
**Job**					
Student	−	−	−	1.00 (Reference)	−
Non-student	−0.30	0.28	−1.09	0.74 (0.43–1.28)	0.277
**Smoke**					
No	−	−	−	1.00 (Reference)	−
Yes	1.19	0.80	1.49	3.30 (0.68–15.91)	0.137
Quit	−1.29	1.16	−1.11	0.27 (0.03–2.69)	0.267
**Alcohol**					
No	−	−	−	1.00 (Reference)	−
Yes	0.06	0.66	0.09	1.06 (0.29–3.88)	0.929
Quit	−2.91	1.05	−2.78	0.05 (0.01–0.42)	**0.005**
**Family stress**					
No	−	−	−	1.00 (Reference)	−
Yes	1.24	0.29	4.27	3.47 (1.96–6.15)	**< 0.001**
**Work stress**					
No	−	−	−	1.00 (Reference)	−
Yes	0.04	0.31	0.13	1.04 (0.57–1.91)	0.897
**Academic stress**					
No	−	−	−	1.00 (Reference)	−
Yes	0.67	0.28	2.43	1.96 (1.14–3.38)	**0.015**
**Interpersonal stress**					
No	−	−	−	1.00 (Reference)	−
Yes	0.85	0.28	3.05	2.34 (1.36–4.03)	**0.002**
**Emotional stress**					
No	−	−	−	1.00 (Reference)	−
Yes	0.21	0.35	0.60	1.23 (0.62–2.43)	0.551
**Financial stress**					
No	−	−	−	1.00 (Reference)	−
Yes	−0.35	0.48	−0.73	0.70 (0.27–1.80)	0.463

Bold values indicate statistical significance at *P* < 0.05.

### 3.3 Sensitivity analysis

To assess the potential influence of sparse-data bias, especially from the small “Quit” subgroups for smoking and alcohol use, we performed a sensitivity analysis. In this analysis, participants originally classified as having “Quit” were recategorized into the larger “No” (never smoked/never drank) reference groups. A multivariable logistic regression model was then refitted using these consolidated variables. The results (Supplementary Table 1) corroborated the primary findings: family stress (adjusted OR = 3.55, 95% CI: 1.85–6.81, *P* < 0.001), interpersonal stress (adjusted OR = 2.12, 95% CI: 1.15–3.92, *P* = 0.017), and BMI (adjusted OR = 0.91, 95% CI: 0.85–0.98, *P* = 0.013) remained independently associated with depression risk. The association for alcohol consumption was altered after reclassification, showing that current drinking was associated with lower odds of depression compared to non-drinking (adjusted OR = 0.19, 95% CI: 0.05–0.69, *P* = 0.011). These findings indicate that the study’s principal conclusions are robust to alternative classifications of these infrequent exposure categories.

### 3.4 Subgroup analysis

To further examine potential variations in the association between psychosocial stress and depression risk across different demographic groups, we performed stratified analyses by age and gender (Supplementary Tables 2, 3). Notably, some effects could not be estimated (denoted as “–”) in certain strata due to complete separation (zero cells).

In the younger group (age ≤ 24 years), family stress, academic stress, and interpersonal stress were all significantly associated with an increased risk of depression (all *P* < 0.05). In the older group (age > 24 years), only family stress remained significantly associated with depression risk (*P* < 0.05), emotional stress was associated with a numerically reduced odds of depression, although this estimate was highly imprecise (with a very wide confidence interval) and should be interpreted with caution (*P* < 0.05).

Among female participants, family stress and BMI were significantly associated with depression risk (both *P* < 0.05). For males, the association between family stress and depression risk did not reach statistical significance (*P* = 0.077); however, a trend toward an increased risk was observed (OR = 3.03, 95% CI: 0.91–10.95).

## 4 Discussion

This cross-sectional study investigated the independent associations of multidimensional psychosocial stress and BMI with depression risk. The results suggest that family stress, academic stress, and interpersonal stress may act as independent risk factors for depression, whereas a higher BMI was associated with a lower risk of depression.

The results show that family stress is a risk factor for depression, which aligns with prior research. Family stress may elevate depression risk by exacerbating parent-child conflict and impairing family functioning (Reck and Kogan, 2021; Luo et al., 2023). Earlier studies have also shown that depressive symptoms among employed married women (Ju et al., 2018) and caregivers of children with chronic illnesses (Khanna et al., 2015) are significantly associated with family stress, consistent with the subgroup findings of this study. Although the association did not reach statistical significance in males, the elevated OR still suggests a potential trend toward increased depression risk, which may be attributable to the limited male sample size and reduced statistical power.

In the exploratory analysis of the older subgroup, the point estimate for emotional stress suggested a negative association with depression risk; however, the small sample size and an imbalanced distribution of depression among those reporting emotional stress resulted in a wide confidence interval, indicating statistical instability. Moreover, academic stress was identified as an independent risk factor in the younger group. Previous studies have indicated that adolescents with academic stress are 2.4 times more likely to develop depression compared to those without (Jayanthi et al., 2015). Academic stress may contribute to depression indirectly by triggering negative emotions and compromising sleep quality (Liu et al., 2023). Individuals under high academic pressure are also more susceptible to school burnout, which may ultimately lead to depressive symptoms (Jiang et al., 2021). In addition, interpersonal stress was associated with depression risk. Chronic negative interpersonal stress can be regarded as a persistent social threat and has been shown to predict depression recurrence (Sheets and Craighead, 2014), possibly through mechanisms involving hypothalamic-pituitary-adrenal axis dysregulation (Oldehinkel and Bouma, 2011). Due to limited subgroup sample sizes, such as individuals reporting financial stress in the younger group, this study could not estimate odds ratios for financial stress. These associations may reflect bidirectional relationships. The stress generation hypothesis suggests that depression may also increase stress exposure or perception (Hammen, 2006; Liu and Alloy, 2010). These findings require validation in larger samples.

These findings may be understood within a neurodevelopmental framework, as adolescence is thought to be a critical period of substantial brain reorganization, particularly within prefrontal-limbic circuits mediating social cognition and emotion regulation. Previous research indicates that environmental stress during this sensitive window may interact with innate neurobiological susceptibility (Fan et al., 2023), while structural neuroimaging studies suggest that early-life stress may influence depression risk through specific neuroanatomical and cellular mechanisms, including alterations in cortical thickness and surface area (Bore et al., 2024). Such mechanisms could potentially underlie the associations observed in the present study, although direct neuroimaging evidence is needed to confirm this hypothesis. Accordingly, future research should consider integrating multidimensional psychosocial stress assessments with structural and functional neuroimaging to investigate whether stress-related brain alterations mediate the associations observed in this study.

Higher BMI was associated with lower depression risk after adjusting for stress, consistent with previous studies (Luo et al., 2018). However, several factors warrant caution in interpreting this finding. First, reverse causality is possible, as depressive symptoms may themselves lead to reduced appetite and weight loss (Treviño-Alvarez et al., 2023). Second, clinical depression is frequently treated with pharmacotherapy, and some antidepressants are known to contribute to weight gain (Serretti and Mandelli, 2010). A paramount confounding factor is the absence of medication data. Consequently, the BMI distribution observed in the depression group may partly reflect treatment-related effects, which could at least partially explain the protective association identified here. Future prospective studies are warranted to systematically collect detailed medication history, including drug type, dosage, and duration, in order to clarify and disentangle the complex causal pathways linking BMI and depression.

The study further identified an association between alcohol abstinence and a lower risk of depression. This finding is consistent with the established view that excessive alcohol consumption is a risk factor for depression (Cho et al., 2024; Visontay et al., 2023). However, the small sample size in the abstinence subgroup (*n* = 14) may have increased the standard error and affected the stability of the estimate. Our sensitivity analysis provided further insight into the complexity of these associations. When former drinkers were merged with never drinkers, current drinking showed an inverse association with depression (OR = 0.19). This finding should be interpreted with extreme caution, as it likely does not imply a protective effect of alcohol, but rather reflects a shift in the composition of the reference group. Therefore, this association warrants further confirmation in studies with larger samples.

Furthermore, the significant difference in alcohol consumption status between the depression and control groups at baseline (Table 1) suggests that the control group may not be fully representative of the general population in terms of lifestyle factors. While we adjusted for alcohol use in our analyses, this difference underscores the importance of considering potential selection bias or broader lifestyle disparities when interpreting comparative findings. The combined “non-current drinking” group may include individuals who abstain due to pre-existing health conditions (including depression itself), thereby altering the baseline risk for comparison. Future studies with larger samples should employ more granular categorization of drinking behavior to clarify these relationships.

This study represents a first attempt to comprehensively assess multidimensional psychosocial stressors, encompassing family, work, financial, academic, emotional, and interpersonal domains, alongside variables such as BMI, smoking, and alcohol use, thereby offering a more holistic perspective on depression risk factors. The statistical approach included multivariable logistic regression to control for potential confounders and subgroup analyses stratified by age and gender, which helped reveal group-specific differences and independent associations. Moreover, despite the limited sample size, the stratified analyses offer preliminary insights into potential variations across age and gender, which may inform the identification of higher-risk populations for future investigation. Collectively, these methodological strengths enhance the credibility of the study’s conclusions and offer a foundation for developing targeted interventions for depression.

Nevertheless, several limitations should be noted. First, the cross-sectional design precludes causal inference. Second, while the overall sample size (*n* = 222) was adequate for primary analyses, it limited statistical power for subgroup examinations. Examining six stress domains alongside multiple covariates also risks overfitting and spurious findings. Therefore, these results require validation in larger prospective studies. Third, the definition of the control group, while standard for community-based cross-sectional studies, relied on self-reported absence of a prior depression diagnosis and a low current BDI-II score, without verification through a structured clinical interview. This approach may not have completely excluded individuals with a past history of depression currently in remission, or those with subclinical symptoms, which could attenuate the observed effect sizes between groups. Fourth, the measurement of psychosocial stress in this study was relatively basic. Stress exposure was assessed using dichotomous (yes/no) items, a method commonly adopted in large-scale epidemiological surveys for practical reasons (Kivimäki et al., 2006), but it fails to capture the intensity, chronicity, frequency, or subjective appraisal of stressors, potentially obscuring dose-response relationships and oversimplifying the complex nexus between stress exposure and depression. Future studies could employ more nuanced and continuous instruments, such as the Perceived Stress Scale (PSS), to better capture the multidimensional nature of stress and its effects on depression.

Fifth, multicollinearity among the six psychosocial stress domains was not formally assessed. Although treated as independent predictors, some domains may conceptually overlap. The potential influence of such intercorrelations on the precision of our estimates remains unquantified. Sixth, BMI was treated as a continuous linear predictor in our models. We acknowledge that the association between BMI and depression may be non-linear, as evidenced by longitudinal studies showing increased risk at both low and high BMI extremes (Jung et al., 2017), and that potential interaction effects between BMI and specific psychosocial stressors remain unexplored. Our cross-sectional design and sample size were not suited to robustly examine such complex relationships. Future prospective studies with larger cohorts should model BMI using categories or flexible non-linear terms to clarify its true association with depression.

Seventh, the absence of medication history data, particularly regarding antidepressants known to influence body weight, represents a significant limitation. This unmeasured confounding factor may have biased the observed association between BMI and depression risk, limiting causal interpretation. Eighth, key clinical characteristics such as age of onset, illness duration, episode number, prior hospitalizations, psychotherapy history, and comorbid disorders were not captured. These unmeasured factors may confound the associations reported herein and limit the clinical interpretability of our findings. Moreover, the absence of structured diagnostic interviews precludes definitive case ascertainment. Future studies should employ standardized diagnostic instruments (such as MINI, SCID-5) and collect comprehensive clinical histories to strengthen validity.

## 5 Conclusion

In summary, this exploratory study simultaneously assessed multiple psychosocial stressors and BMI in relation to depression risk. Family, academic, and interpersonal stress emerged as consistent independent risk factors, with notable variations by age and gender, whereas work, financial, and emotional stress showed no significant associations. The observed inverse association between higher BMI and depression risk should be interpreted cautiously given potential unmeasured confounders such as antidepressant use. These findings help prioritize key modifiable stressors for future longitudinal research and targeted intervention development. Future studies should employ prospective designs, larger cohorts, and more granular stress measurements to validate these preliminary observations and clarify causal pathways.

## Data Availability

The raw data supporting the conclusions of this article will be made available by the authors, without undue reservation.
