# Supplementary material for: A cross-sectional study of multidimensional psychosocial stress and depression risk
Source: Front Behav Neurosci. 2026 Mar 12;20:1786960. doi: 10.3389/fnbeh.2026.1786960 (PMC13018114; doi:10.3389/fnbeh.2026.1786960)
Supplement: Supplementary file 1 [file Data_Sheet_1.docx]

**Supplementary Tables**

**Table 1. Multivariable Logistic Regression Analysis with Consolidated Variables**

| Variables | β | S.E | Z | OR (95% CI) | P |
| --- | --- | --- | --- | --- | --- |
|  |  |  |  |  |  |
| Intercept | 0.03 | 1.17 | 0.03 | 1.03 (0.11–10.17) | 0.977 |
| Age | 0.03 | 0.02 | 1.39 | 1.03 (0.99–1.08) | 0.163 |
| BMI | -0.09 | 0.04 | -2.49 | 0.91 (0.85–0.98) | **0.013** |
| Gender |  |  |  |  |  |
| Female |  |  |  | 1.00 (Reference) |  |
| Male | -0.24 | 0.34 | -0.70 | 0.79 (0.40–1.54) | 0.485 |
| Job |  |  |  |  |  |
| Non-student |  |  |  | 1.00 (Reference) |  |
| Student | 0.59 | 0.54 | 1.11 | 1.81 (0.63–5.18) | 0.269 |
| Smoke |  |  |  |  |  |
| No |  |  |  | 1.00 (Reference) |  |
| Yes | 1.68 | 0.86 | 1.94 | 5.36 (0.98–29.18) | 0.052 |
| Alcohol |  |  |  |  |  |
| No |  |  |  | 1.00 (Reference) |  |
| Yes | -1.66 | 0.65 | -2.53 | 0.19 (0.05–0.69) | **0.011** |
| Family stress |  |  |  |  |  |
| No |  |  |  | 1.00 (Reference) |  |
| Yes | 1.27 | 0.33 | 3.82 | 3.55 (1.85–6.81) | **<.001** |
| Work stress |  |  |  |  |  |
| No |  |  |  | 1.00 (Reference) |  |
| Yes | 0.48 | 0.44 | 1.10 | 1.62 (0.68–3.86) | 0.272 |
| Academic stress |  |  |  |  |  |
| No |  |  |  | 1.00 (Reference) |  |
| Yes | 0.55 | 0.39 | 1.42 | 1.73 (0.81–3.70) | 0.156 |
| Interpersonal stress |  |  |  |  |  |
| No |  |  |  | 1.00 (Reference) |  |
| Yes | 0.75 | 0.31 | 2.40 | 2.12 (1.15–3.92) | **0.017** |
| Emotional stress |  |  |  |  |  |
| No |  |  |  | 1.00 (Reference) |  |
| Yes | -0.19 | 0.44 | -0.44 | 0.83 (0.35–1.94) | 0.662 |
| Financial stress |  |  |  |  |  |
| No |  |  |  | 1.00 (Reference) |  |
| Yes | -0.05 | 0.60 | -0.07 | 0.96 (0.29–3.13) | 0.941 |

**Table 2. Multivariate Logistic Regression Analysis Stratified by Gender**

| **Variables** | **Male (n = 75)** | | **Female (n = 147)** | |
| --- | --- | --- | --- | --- |
|  | **OR (95% CI)** | **P** | **OR (95% CI)** | **P** |
| Age | 1.02 (0.93–1.12) | 0.721 | 1.03 (0.97–1.09) | 0.377 |
| BMI | 0.92 (0.79–1.02) | 0.176 | 0.90 (0.81–0.99) | 0.033 |
| Job |  |  |  |  |
| Student | Ref | Ref | Ref | Ref |
| Non-student | 2.26 (0.16–35.35) | 0.547 | 0.51 (0.14–1.79) | 0.291 |
| Family stress |  |  |  |  |
| No | Ref | Ref | Ref | Ref |
| Yes | 3.03 (0.91–10.95) | 0.077 | 3.49 (1.54–8.38) | 0.004 |
| Work stress |  |  |  |  |
| No | Ref | Ref | Ref | Ref |
| Yes | 2.11 (0.41–12.43) | 0.380 | 1.23 (0.44–3.57) | 0.690 |
| Academic stress |  |  |  |  |
| No | Ref | Ref | Ref | Ref |
| Yes | 2.84 (0.55–17.88) | 0.229 | 1.67 (0.68–4.15) | 0.264 |
| Interpersonal stress |  |  |  |  |
| No | Ref | Ref | Ref | Ref |
| Yes | 1.60 (0.50–5.17) | 0.423 | 2.04 (0.96–4.42) | 0.067 |
| Emotional stress |  |  |  |  |
| No | Ref | Ref | Ref | Ref |
| Yes | 0.27 (0.04–1.32) | 0.121 | 1.30 (0.42–4.29) | 0.654 |
| Financial stress |  |  |  |  |
| No | Ref | Ref | Ref | Ref |
| Yes | 0.61 (0.05–6.52) | 0.683 | 0.78 (0.16–3.81) | 0.758 |

**Table 3. Multivariate Logistic Regression Analysis Stratified by Age**

| **Variables** | **Age≤24 years (n = 158)** | | **Age＞24 years (n = 64)** | |
| --- | --- | --- | --- | --- |
|  | **OR (95% CI)** | **P** | **OR (95% CI)** | **P** |
| BMI | 0.92 (0.84–0.99) | 0.051 | 0.95 (0.73–1.22) | 0.687 |
| Gender |  |  |  |  |
| Male | Ref | Ref | Ref | Ref |
| Female | 1.86 (0.81–4.36) | 0.146 | 0.56 (0.10–2.91) | 0.500 |
| Job |  |  |  |  |
| Student | Ref | Ref | Ref | Ref |
| Not-student | 0.82 (0.21–3.35) | 0.782 | 2.76 (0.04–244.17) | 0.641 |
| Family stress |  |  |  |  |
| No | Ref | Ref | Ref | Ref |
| Yes | 3.06(1.36–7.21) | 0.008 | 7.39 (1.70–42.90) | 0.013 |
| Work stress |  |  |  |  |
| No | Ref | Ref | Ref | Ref |
| Yes | 2.37 (0.63–10.91) | 0.226 | 0.60 (0.13–2.41) | 0.479 |
| Academic stress |  |  |  |  |
| No | Ref | Ref | Ref | Ref |
| Yes | 3.21 (1.26–8.57) | 0.016 | 0.39 (0.04–2.86) | 0.364 |
| Interpersonal stress |  |  |  |  |
| No | Ref | Ref | Ref | Ref |
| Yes | 2.62 (1.23–5.73) | 0.013 | 1.07 (0.22–5.14) | 0.935 |
| Emotional stress |  |  |  |  |
| No | Ref | Ref | Ref | Ref |
| Yes | 1.10 (0.36–3.60) | 0.873 | 0.04 (0.00–0.56) | 0.033 |
| Financial stress |  |  |  |  |
| No | Ref | Ref | Ref | Ref |
| Yes | - | 0.986 | 0.30 (0.03–2.15) | 0.259 |
